# Comparison of Manual Multi-ROI, Single-Location, and Volumetric Fat Fraction Measurements for Hepatic Steatosis Using MRI

**DOI:** 10.3390/diagnostics16050716

**Published:** 2026-02-27

**Authors:** Nurullah Dag, Muhammed Kürşat Bilaloğlu, Ramazan Kutlu

**Affiliations:** Department of Radiology, Faculty of Medicine, Inonu University, Malatya 44280, Türkiye; kursatbilaloglu@gmail.com (M.K.B.); ramazan.kutlu@inonu.edu.tr (R.K.)

**Keywords:** liver fat quantification, hepatic steatosis, magnetic resonance imaging, proton density fat fraction, magnetic resonance spectroscopy

## Abstract

**Background/Objectives:** To comparatively evaluate the clinical reliability of automated single-location MRI–proton density fat fraction (PDFF), single-voxel magnetic resonance spectroscopy (MRS), and volumetric MRI-PDFF measurements for quantitative assessment of hepatic steatosis, using manual multi-regions of interest (ROI) MRI-PDFF as the reference standard. **Methods:** In this retrospective single-center study, adult patients who underwent liver MRI with both MRI-PDFF and MRS between December 2024 and January 2026 were included. Manual multi-ROI MRI-PDFF measurements were obtained from eight Couinaud segments and served as the reference standard. Automated single-location MRI-PDFF, single-voxel MRS, and volumetric MRI-PDFF measurements were extracted from system-generated reports. Fat fraction values and steatosis grades were compared using non-parametric tests, Spearman correlation coefficients, exact agreement rates, weighted Cohen’s kappa statistics, and Bland–Altman analysis. Intra- and inter-observer reliability of manual multi-ROI measurements was assessed using intraclass correlation coefficients (ICC). **Results:** A total of 490 patients were included. Single-location MRI-PDFF and single-voxel MRS demonstrated high exact agreement with manual multi-ROI MRI-PDFF in steatosis grading (86.9% and 87.4%, respectively), with near-perfect agreement (weighted κ = 0.82–0.83). Volumetric MRI-PDFF showed lower exact agreement (76.1%) and a systematic tendency toward overestimation, with more frequent upward shifts in steatosis grade. All techniques showed strong correlations with manual measurements (ρ = 0.72–0.75). Manual multi-ROI MRI-PDFF demonstrated excellent inter- and intra-observer reliability (ICC > 0.97). **Conclusions:** Automated single-ROI MRI-PDFF and single-voxel MRS provide clinically reliable and time-efficient alternatives for hepatic steatosis assessment, while automated volumetric MRI-PDFF may introduce systematic bias toward overestimation.

## 1. Introduction

Metabolic dysfunction-associated steatotic liver disease (MASLD) has emerged as one of the most prevalent chronic liver disorders worldwide and represents a major public health challenge [1]. Closely linked to obesity, insulin resistance, and type 2 diabetes mellitus, MASLD is estimated to affect approximately 25–30% of the adult population globally, reflecting the hepatic manifestation of systemic metabolic dysfunction [2,3]. Hepatic steatosis, characterised by the accumulation of triglycerides in excess (>5%) within hepatocytes, constitutes the earliest and most prevalent feature of MASLD and is frequently observed in both asymptomatic individuals and patients with established metabolic disease [4,5]. While simple steatosis may remain stable over time in a substantial proportion of affected individuals, a subset of patients develops metabolic dysfunction–associated steatohepatitis, a more aggressive phenotype characterised by hepatocellular injury and inflammation, which in turn increases the risk of fibrosis progression, cirrhosis, and hepatocellular carcinoma [5,6].

In view of the high prevalence of MASLD in the general population and its potential for progressive liver injury in selected patients, the accurate and reproducible quantification of hepatic fat content has become increasingly important. Reliable assessment of liver fat is essential not only for disease detection and risk stratification but also for longitudinal monitoring and patient selection in clinical trials targeting metabolic liver disease [4,7]. Although liver biopsy has historically served as the reference standard for steatosis assessment, its invasive nature, limited feasibility for repeated measurements, and well-documented sampling variability—particularly in the setting of heterogeneous fat distribution—restrict its routine clinical and research utility [8]. These limitations have given rise to a growing interest in noninvasive imaging-based techniques capable of providing quantitative and whole-liver evaluation of hepatic fat content [9].

Magnetic resonance imaging (MRI) is currently considered one of the most reliable methods for non-invasive and quantitative assessment of tissue fat content due to its lack of ionising radiation, high soft tissue contrast, and ability to distinguish biochemical components [10,11]. In fat quantification with MRI, magnetic resonance spectroscopy (MRS) and chemical shift-based MRI-proton density fat fraction (PDFF) techniques are primarily used. MRS has long been accepted the reference standard due to its direct measurement of fat fraction based on the spectral separation of water and fat protons within a given volumetric voxel, and has shown high correlation with histopathology [11,12]. However, its disadvantages include limited sampling volume, an inability to reflect regional heterogeneity and technical complexity, which constitute limitations [13]. To overcome these limitations, MRI-PDFF techniques have been developed, employing multiple echo chemical shift-encoded approaches to correct for T1 bias, T2* effects, spectral fat complexity, and magnetic field inhomogeneities. Thus, it provides voxel-based, whole-organ volume, repeatable and platform-independent fat fraction measurement [11,12]. Multicenter and longitudinal studies have shown that MRI-PDFF measurements correlate strongly with MRS, offer high inter-observer and inter-instrument agreement, and can reliably detect even small changes in fat content [12,13,14]. Given these technical advantages, MRI-PDFF is considered the gold standard imaging-based method for evaluating fatty liver disease in the current literature [14,15].

The aim of this study is to comparatively evaluate the clinical reliability of automated single-location MRI–PDFF, single-voxel MRS, and volumetric MRI-PDFF measurements for the quantitative assessment of hepatic steatosis using MRI, with manual multi-regions of interest (ROI) MRI-PDFF as the reference standard. The study further examines whether automated single-location and volumetric measurements introduce systematic bias in steatosis assessment and evaluates the intra- and inter-observer reliability of manual multi-ROI PDFF measurements.

## 2. Materials and Methods

### 2.1. Patient Selection

This single-center, analytical, retrospective study evaluated all adult patients (≥18 years) who underwent liver MRI between December 2024 and January 2026. Patients were eligible for inclusion if hepatic fat quantification had been performed using both MRI-PDFF and MRS, enabling direct comparison between quantitative techniques.

**Figure 1:**
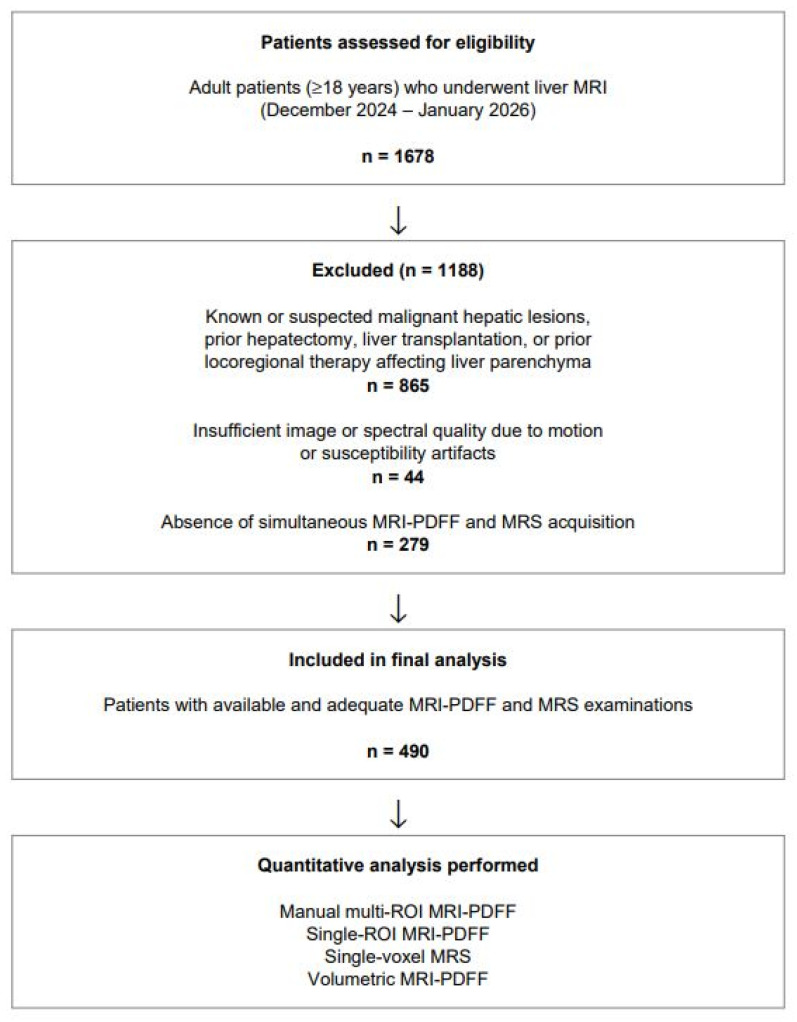
Flowchart illustrating patient selection and exclusion criteria.

Patients with known or suspected malignant hepatic lesions, those who had undergone prior locoregional therapies, hepatectomy or liver transplant, and individuals with benign liver lesions extending across multiple segments were excluded to avoid potential confounding effects on parenchymal fat measurements. Additionally, examinations with insufficient image or spectral quality due to motion or susceptibility artifacts were excluded, as reliable fat quantification requires adequate signal-to-noise ratio and artifact-free acquisitions. Patients in whom one of the quantitative techniques was unavailable were also excluded. The patient selection process and reasons for exclusion are summarized in the study flowchart (Figure 1).

### 2.2. Magnetic Resonance Imaging Protocol

All examinations were performed on a 1.5-T MRI scanner (MAGNETOM Altea, Siemens Healthcare, Erlangen, Germany) equipped with a 32-channel body matrix coil. MRI-PDFF was acquired in the axial plane using the breath-hold, six-point QDixon method. The imaging parameters were repetition time (TR), 9.0 ms; echo time (TE), 1.05, 2.46, 3.69, 4.92, 6.15, and 7.38 ms; field of view, 450 × 450 mm; slice thickness, 3.5 mm; flip angle, 40°; bandwidth, 1080 Hz/pixel.

MRS was acquired with the HISTO sequence (High-speed T2-corrected multi-echo spectroscopy). Spectra were acquired using a STEAM localization technique without water suppression. The acquisition parameters were as follows: repetition time (TR), 3000 ms; echo times (TEs), 12, 24, 36, 48, and 72 ms; flip angle, 90°; spectral bandwidth, approximately 1200 Hz; number of data points, 1024; and number of signal averages, 1. Data acquisition was performed during breath-hold. Automatic T2 correction for both water and fat signals and a multi-peak fat spectral model were applied for inline calculation of the fat fraction.

### 2.3. Image Analysis and Fat Fraction Measurements

Manual multi-ROI MRI-PDFF measurements were obtained by placing circular ROIs with an area of 3 cm^2^ in each of the eight Couinaud liver segments, and the mean value of these measurements was recorded as the representative hepatic fat fraction. This approach was selected as the reference standard because multi-segment sampling provides a more representative assessment of whole-liver fat distribution. All measurements were performed independently by two radiologists with approximately 11 and 2 years of experience in abdominal imaging, who were blinded to the patients’ clinical information and to each other’s measurements. To assess intra- and interobserver reliability, approximately 10% of randomly selected participants were re-evaluated in a separate session, with both readers blinded to the initial measurements.

**Figure 2:**
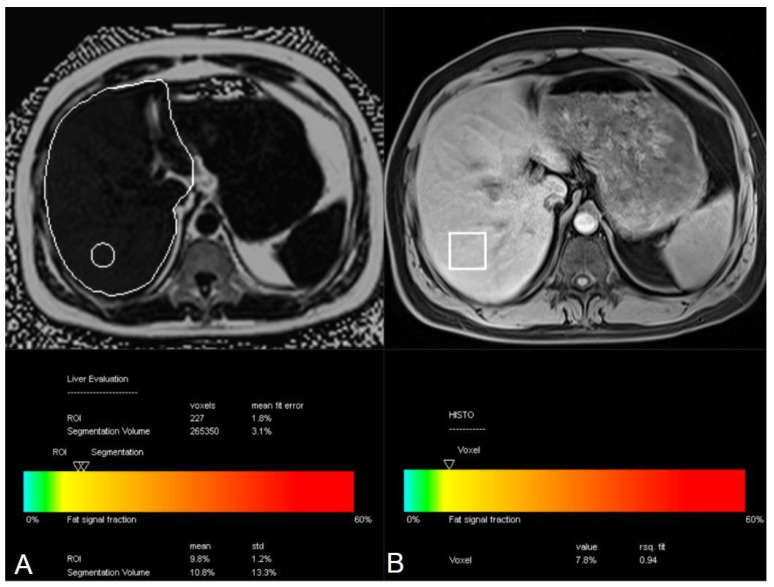
(**A**) Single-ROI and volumetric MRI–PDFF measurements obtained from the right hepatic lobe. In the automated whole-liver segmentation, contour spillover beyond the hepatic capsule into adjacent mesenteric fat should be carefully noted, as this may lead to overestimation of volumetric fat fraction values. (**B**) Single-voxel MRS acquisition positioned within the right hepatic lobe.

Single-location measurements, defined as a single technologist-placed sampling region, were obtained using both MRI-PDFF and MRS techniques based on sampling regions placed by the technologist during image acquisition. For MRI-PDFF, an oval ROI with an area of 3 cm^2^ was positioned in the right hepatic lobe. For MRS, a single cubic voxel with a typical size of 30 × 30 × 30 mm^3^ was placed in the right hepatic lobe during acquisition. Volumetric fat fraction measurements were automatically generated by the vendor-provided software using whole-liver segmentation of the MRI-PDFF dataset. No manual contour editing, post-processing correction, or exclusion of vascular or biliary structures was performed. Fat fraction values derived from all three approaches were obtained from the system-generated reports. Representative examples of ROI placement, voxel positioning, and automated report outputs for each quantitative technique are illustrated in Figure 2.

For all measurement techniques, ROIs and voxels were carefully positioned at least 1–2 cm away from major vascular structures, bile ducts, and the hepatic capsule, in accordance with established recommendations aimed at minimizing partial volume effects and susceptibility-related inaccuracies in hepatic fat quantification.

Based on fat fraction values, hepatic steatosis was classified as normal (S0, <6%), mild (S1, 6–17%), moderate (S2, 17–22%), and severe (S3, >22%) [16].

### 2.4. Ethical Approval and Funding

The study was performed in compliance with the ethical standards outlined in the Declaration of Helsinki and its later revisions, as well as relevant institutional and national regulations governing research involving human subjects. Approval for this retrospective study was granted by the Inonu University Institutional Review Board for Non-Interventional Studies (Approval No: 2025/8755). Given the retrospective nature of the study and the use of previously acquired clinical and imaging data, the requirement for written informed consent was waived by the ethics committee.

Financial support for this research was provided by the Inonu University Scientific Research Projects Coordination Unit (Grant No: 2026/4500). The study was designed, conducted, and reported in accordance with the Strengthening the Reporting of Observational Studies in Epidemiology (STROBE) recommendations to enhance transparency, methodological quality, and reproducibility [17].

### 2.5. Statistical Analysis

Statistical analyses were performed using appropriate non-parametric and agreement-based methods due to the non-normal distribution of fat fraction measurements. Continuous variables were summarized as mean ± standard deviation or median with interquartile range (IQR), as appropriate, while categorical variables were expressed as frequencies and percentages. Comparisons between fat fraction values obtained using different quantitative techniques were conducted using paired non-parametric tests, with manual multi-ROI fat fraction measurements serving as the reference standard. Correlations between measurement methods were assessed using Spearman’s rank correlation coefficient (ρ).

Agreement between steatosis grades derived from different methods was evaluated using exact agreement rates and weighted Cohen’s kappa (κ) statistics, with kappa values interpreted according to standard benchmarks (poor, fair, moderate, substantial, and excellent agreement). Non-adjacent class shifts were specifically analyzed to identify clinically relevant misclassification across steatosis grades. Agreement between quantitative measurements was further examined using Bland–Altman analysis, reporting mean bias and 95% limits of agreement (LoA).

Interobserver and intraobserver reliability for manual fat fraction measurements were assessed using intraclass correlation coefficients (ICC) with 95% confidence intervals. A two-sided *p*-value < 0.05 was considered statistically significant for all analyses.

## 3. Results

### 3.1. Study Population Characteristics

A total of 490 adult patients were included in the final analysis. The mean age was 52.6 ± 15.4 years (range, 18–90 years), with an approximately equal sex distribution (49.8% female, 50.2% male). The median body mass index was 25.86 kg/m^2^ (IQR, 24.22–28.67). Based on BMI classification, 42.7% of participants had normal weight, 42.2% were overweight, and 15.1% were obese (Table 1).

### 3.2. Comparison of Fat Fraction Measurements Across Methods

**Figure 3:**
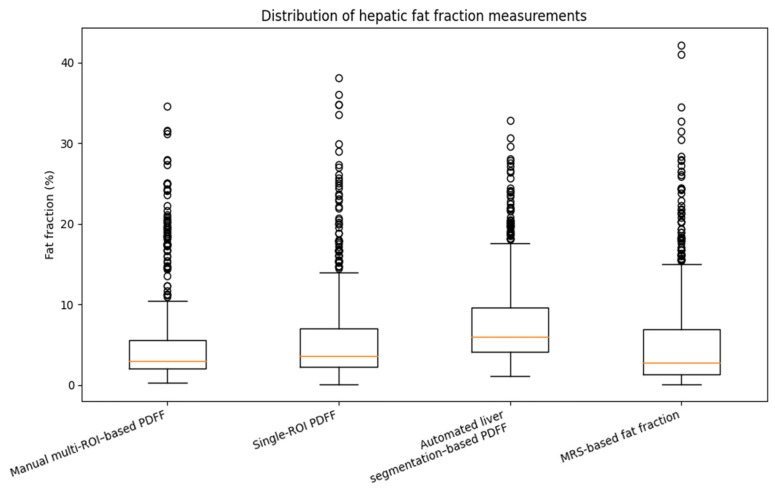
Comparison of hepatic fat fraction values obtained using manual multi-ROI MRI–PDFF, single-ROI MRI–PDFF, volumetric MRI–PDFF, and single-voxel MRS measurements.

According to the MRI-PDFF–based steatosis grading, 279 patients (56.9%) were classified as S0, 135 (27.5%) as S1, 47 (9.5%) as S2, and 29 (5.9%) as S3. Median fat fraction values differed significantly across measurement techniques (Table 2 and Figure 3). Manual multi-ROI MRI-PDFF measurements, used as the reference standard, yielded a median fat fraction of 2.95% (IQR, 2.00–5.50). Single-ROI MRI-PDFF measurements demonstrated a modest but statistically significant increase compared with manual measurements (median difference, +0.20%, *p* < 0.001). In contrast, volumetric MRI-PDFF showed a markedly higher median fat fraction (6.00%, IQR, 4.10–9.63), corresponding to a median overestimation of +1.90% relative to manual measurements (*p* < 0.001). Single-voxel MRS-based fat fraction measurements were slightly lower than manual values, with a median difference of −0.70% (*p* < 0.001).

### 3.3. Agreement in Steatosis Grading

Exact agreement in steatosis grade classification was highest for single-voxel MRS-based fat fraction (87.4%) and single-ROI MRI-PDFF (86.9%), both demonstrating excellent agreement with manual measurements (weighted κ = 0.83 and 0.82, respectively). Volumetric MRI-PDFF showed lower exact agreement (76.1%) and substantial agreement (weighted κ = 0.68), with a predominant tendency toward overestimation, particularly transitions from S1 to S2 and S2 to S3. Non-adjacent class shifts were rare for single-ROI MRI-PDFF and single-voxel MRS measurements (<1%) but were more frequent with volumetric MRI-PDFF (3–5%) (Table 3).

### 3.4. Correlation Between the Techniques

**Figure 4:**
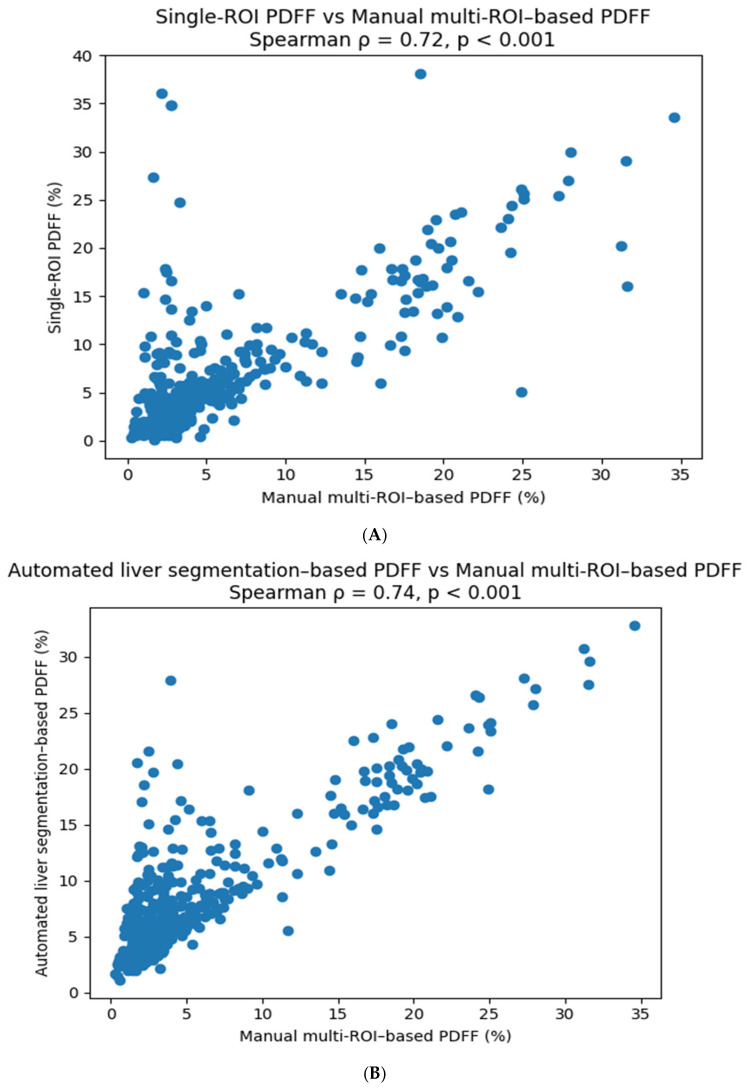

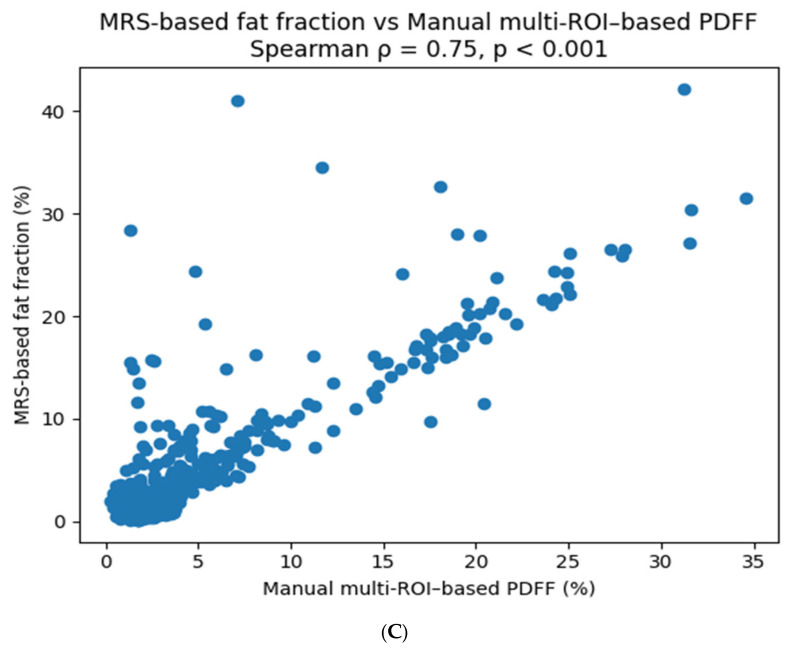
(**A**) Correlation between manual multi-ROI MRI–PDFF and single-ROI MRI–PDFF measurements. (**B**) Correlation between manual multi-ROI MRI–PDFF and volumetric MRI–PDFF measurements. (**C**) Correlation between manual multi-ROI MRI–PDFF and single-voxel MRS measurements.

All quantitative methods demonstrated strong positive correlations with manual multi-ROI MRI-PDFF measurements. Spearman correlation coefficients were ρ = 0.72 for single-ROI MRI-PDFF, ρ = 0.74 for volumetric MRI-PDFF, and ρ = 0.75 for single-voxel MRS-based measurements (all *p* < 0.001) (Table 4, Figure 4). BMI category showed a statistically significant but weak effect on the disagreement between single-ROI and manual multi-ROI measurements (*p* < 0.05), whereas volumetric MRI-PDFF and single-voxel MRS measurements were not significantly influenced by BMI category (*p* > 0.05).

### 3.5. Bland–Altman Agreement Analysis

**Figure 5:**
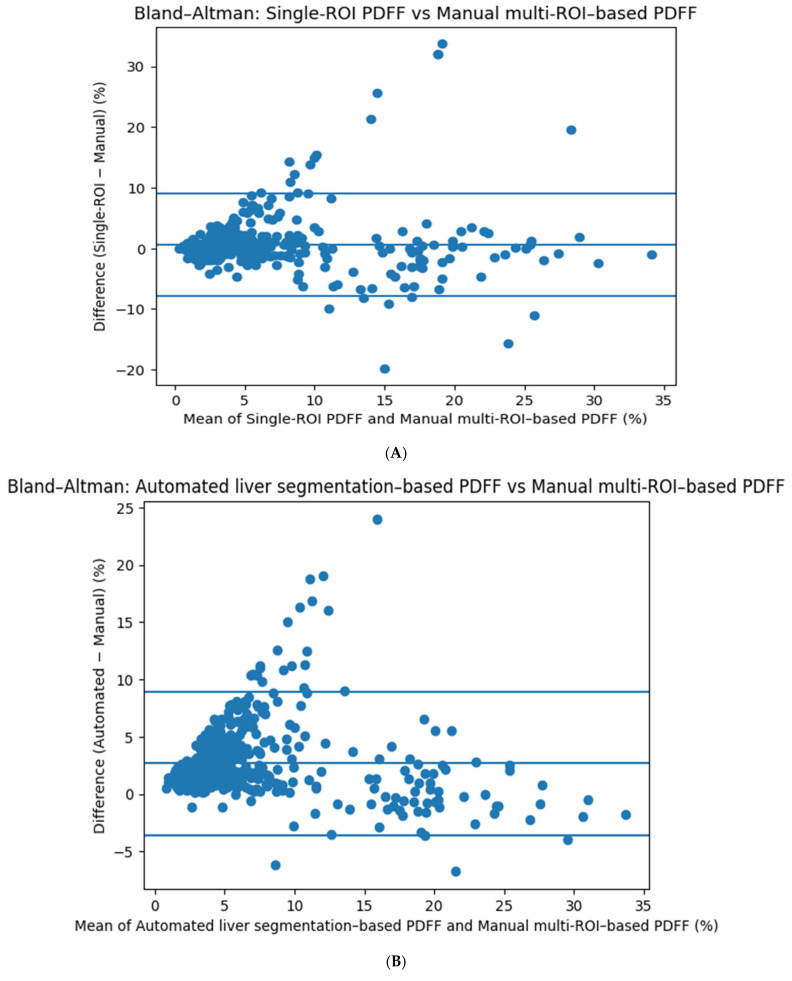

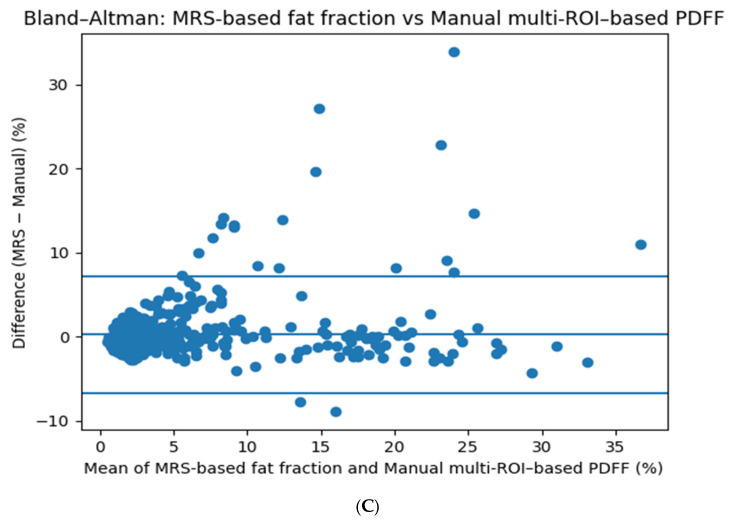
(**A**) Bland–Altman plot demonstrating agreement between manual multi-ROI MRI–PDFF and single-ROI MRI–PDFF measurements. (**B**) Bland–Altman plot demonstrating agreement between manual multi-ROI MRI–PDFF and volumetric MRI–PDFF measurements. (**C**) Bland–Altman plot demonstrating agreement between manual multi-ROI MRI–PDFF and single-voxel MRS measurements.

Bland–Altman analysis demonstrated good agreement between single-ROI MRI-PDFF and manual multi-ROI MRI-PDFF measurements, with a low mean bias of approximately +0.6–0.8% and moderately wide limits of agreement (Figure 5A). Volumetric MRI-PDFF exhibited a higher positive bias (~+2.7%), indicating systematic overestimation, particularly at higher fat fraction values (Figure 5B). In contrast, single-voxel MRS-based measurements showed minimal bias (~+0.2%) and narrower limits of agreement, reflecting closer alignment with manual multi-ROI MRI-PDFF measurements across the full range of fat fraction values (Figure 5C).

### 3.6. Interobserver and Intraobserver Reliability

**Figure 6:**
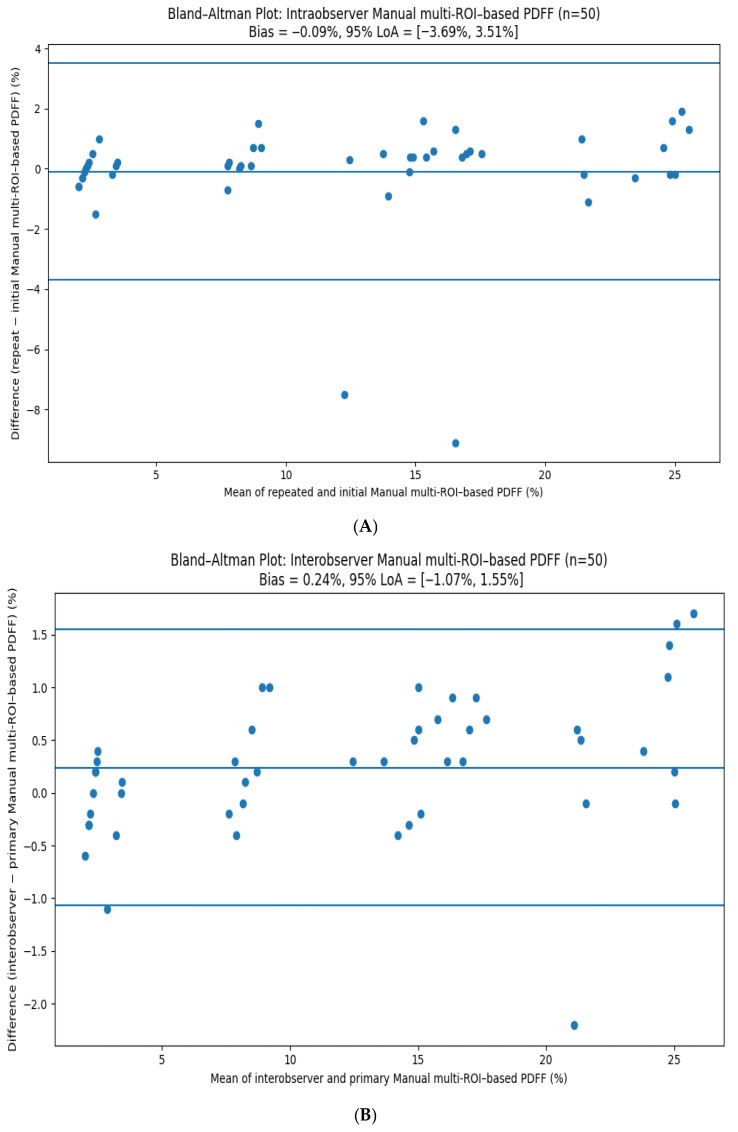
(**A**) Bland–Altman analysis of interobserver agreement for manual multi-ROI MRI–PDFF measurements. (**B**) Bland–Altman analysis of intraobserver agreement for manual multi-ROI MRI–PDFF measurements.

Manual multi-ROI MRI-PDFF measurements demonstrated excellent reproducibility. Interobserver reliability was high, with an ICC of 0.982 (95% CI, 0.972–0.989), while intraobserver reliability was similarly strong, with an ICC of 0.974 (95% CI, 0.955–0.985). Bland–Altman analyses confirmed minimal measurement bias and narrow limits of agreement for both inter- and intraobserver comparisons (Figure 6).

## 4. Discussion

In this study, single-location MRI–PDFF and single-voxel MRS measurements automatically reported by the scanner demonstrated high concordance in steatosis grading when compared with the manual multi-ROI MRI-PDFF reference. The high exact agreement rates and near-perfect weighted kappa values observed for both techniques indicate that they may serve as time-efficient and clinically reliable alternatives in routine practice. These results are in line with prior studies demonstrating strong agreement between MRI-PDFF, MRS, and histopathologic fat quantification [11,12,13,18]. Nevertheless, because single-location approaches sample a limited anatomical volume, their accuracy may be reduced in the presence of heterogeneous hepatic fat distribution. Indeed, previously reported inter-lobar and segmental variations in PDFF suggest that such measurements may not fully capture the global hepatic fat burden [19]. Manual multi-ROI MRI-PDFF was selected as the reference standard in this study because averaging measurements across multiple Couinaud segments provides a more representative estimate of global hepatic fat content and mitigates the impact of regional fat heterogeneity. Although single-voxel MRS is widely regarded as a technical reference for fat quantification, its limited sampling volume restricts its ability to reflect whole-liver fat distribution [20,21]. Therefore, a multi-segment MRI-PDFF approach was considered more appropriate as an internal reference standard for comparative analysis of clinically used measurement strategies.

Notably, automated volumetric MRI-PDFF measurements in our study consistently yielded higher fat fraction values than manual multi-ROI measurements and showed a tendency toward upward shifts in steatosis grade classification. This finding indicates that, despite strong correlation coefficients, volumetric PDFF may introduce systematic bias in clinical staging. Although earlier studies have reported high reproducibility and strong correlations for automated whole-liver PDFF measurements [22,23,24,25], these techniques inherently include regions adjacent to intrahepatic vessels, biliary structures, and the hepatic capsule, which may influence mean PDFF values [23,25]. The more frequent S1-to-S2 and S2-to-S3 transitions observed with volumetric PDFF in our cohort underscore this methodological effect and further support the notion that high correlation does not necessarily equate to clinical interchangeability. A weak BMI-related variability was observed for single-ROI measurements; however, this finding likely reflects the inherent susceptibility of single-location sampling to local heterogeneity rather than a clinically meaningful biologic effect.

The exceptionally high intra- and inter-observer reliability observed for manual multi-ROI MRI–PDFF measurements in our study underscores the methodological robustness of this approach. Averaging measurements across eight Couinaud segments yields a more representative estimate of global hepatic fat content by mitigating the effects of regional heterogeneity in intrahepatic fat distribution. These findings are consistent with prior methodological studies demonstrating that the use of multiple, large ROIs is associated with narrower limits of agreement and improved reproducibility [18,26]. Moreover, the high intraclass correlation coefficients achieved between observers with differing levels of experience suggest that the multi-ROI strategy minimizes user dependence and provides a reliable measurement standard for both clinical trials and reference-based clinical applications.

Recent systematic reviews have emphasized that the selection of MRI-based techniques for hepatic fat quantification should be driven not only by correlation metrics but also by robustness against sampling bias and clinical interpretability [27,28]. Clinical imaging studies have further highlighted that automatically generated PDFF values should be interpreted with caution when used for clinical decision-making, particularly near diagnostic thresholds, as methodological differences between sampling strategies may influence steatosis classification [27,29,30]. Importantly, multi-center and multi-vendor validation studies have demonstrated excellent technical reproducibility of MRI-PDFF across scanners and field strengths when standardized protocols are applied, indicating that discrepancies observed between measurement approaches primarily reflect intrinsic methodological differences rather than hardware-related variability [31]. Comparative analyses of ROI-based and whole-liver strategies have reinforced that multi-segment ROI sampling offers a favorable balance between representativeness and susceptibility to bias in heterogeneous steatosis [30,32]. In addition, studies comparing MRI-PDFF with MRS have underscored that sampling strategy plays a critical role in accurate fat quantification, supporting the continued use of multi-ROI approaches as a methodological reference standard [33].

This study has several limitations. First, its retrospective and single-center design may limit the generalizability of the findings to other institutions and imaging platforms. Second, the predominance of patients with normal or low steatosis in our cohort may have reduced sensitivity for detecting discrepancies at higher fat fractions and may limit the generalizability of our findings to patients with clinically significant MASLD. Third, although manual multi-ROI MRI-PDFF was used as the reference standard, histopathologic correlation was not available, precluding direct validation against liver biopsy. However, MRI-PDFF has been extensively validated in prior studies as a reliable noninvasive surrogate for hepatic fat quantification. Fourth, all MRI examinations were performed on a single 1.5-T scanner from one vendor; therefore, the impact of different field strengths and scanner platforms on measurement discrepancies could not be directly assessed. Fifth, automated single-location measurements were placed by technologists during image acquisition, which may introduce variability related to voxel or ROI positioning. Finally, volumetric PDFF measurements were derived from vendor-provided software, and the findings may not be directly applicable to alternative segmentation algorithms or post-processing pipelines.

## 5. Conclusions

In conclusion, this study demonstrates that automated single-ROI MRI-PDFF and single-voxel MRS measurements show high agreement with manual multi-ROI MRI-PDFF and can be considered practical and reliable tools for routine hepatic steatosis assessment. In contrast, automated volumetric MRI-PDFF measurements tend to overestimate hepatic fat content and may lead to upward shifts in steatosis grading, particularly near diagnostic thresholds. Manual multi-ROI MRI-PDFF provides excellent reproducibility and mitigates the effects of regional fat heterogeneity, supporting its role as a robust reference standard in diagnostic imaging, clinical trials, and longitudinal disease monitoring. These findings underscore the importance of considering measurement strategy and sampling methodology when interpreting automated PDFF reports in clinical practice.

## Figures and Tables

**Table 1:** Demographic and clinical characteristics of the study population.

Variable	Value (*n* = 490)
Age (years), mean ± SD (range)	52.6 ± 15.4 (18–90)
Sex, *n* (%)	
–Female	244 (49.8)
–Male	246 (50.2)
Height (cm), mean ± SD (range)	169.1 ± 7.2 (110–192)
Weight (kg), mean ± SD (range)	75.5 ± 11.3 (45–130)
Body mass index (kg/m^2^), median (IQR)	25.86 (24.22–28.67)
BMI categories, *n* (%)	
–Normal weight	209 (42.7)
–Overweight	207 (42.2)
–Obese	74 (15.1)

**Table 2:** Comparison of hepatic fat fraction values across different quantitative measurement techniques.

Measurement Method	Median	IQR	Median Difference	*p*-Value
manual multi-ROI MRI-PDFF	2.95	2.00–5.50	–	–
single-ROI MRI-PDFF	3.60	2.20–7.05	+0.20	<0.001
volumetric MRI-PDFF	6.00	4.10–9.63	+1.90	<0.001
single-voxel MRS	2.80	1.30–6.90	−0.70	<0.001

**Table 3:** Agreement in steatosis grade classification between manual multi-ROI MRI–PDFF and alternative measurement methods.

Measurement Method	Exact Agreement (%)	Weighted κ (95% CI)	Predominant Direction of Disagreement	Non-Adjacent Class Shift (%)
single-ROI MRI-PDFF	86.9	0.82 (excellent)	S0 → S1, S1 → S2	<1%
volumetric MRI-PDFF	76.1	0.68 (substantial)	S1 → S2, S2 → S3 (overestimation)	3–5%
single-voxel MRS	87.4	0.83 (excellent)	S0 → S1, S1 → S2	<1%

**Table 4:** Spearman correlation coefficients between manual multi-ROI MRI–PDFF and other quantitative techniques.

Comparison	Spearman’s ρ	*p*-Value
manual multi-ROI MRI-PDFF vs. single-ROI MRI-PDFF	0.72	<0.001
manual multi-ROI MRI-PDFF vs. volumetric MRI-PDFF	0.74	<0.001
manual multi-ROI MRI-PDFF vs. single-voxel MRS	0.75	<0.001

## Data Availability

The datasets analyzed during the current study are available from the corresponding author on reasonable request.

## Abbreviations

BMI	Body Mass Index
CI	Confidence Interval
ICC	Intraclass Correlation Coefficient
IQR	Interquartile Range
κ (Kappa)	Cohen’s Kappa Coefficient
LoA	Limits of Agreement
MASLD	Metabolic Dysfunction-Associated Steatotic Liver Disease
MRI	Magnetic Resonance Imaging
MRI-PDFF	Magnetic Resonance Imaging–Proton Density Fat Fraction
MRS	Magnetic Resonance Spectroscopy
PDFF	Proton Density Fat Fraction
QDixon	Quantitative Dixon
ROI	Region of Interest
S0–S3	Steatosis Grades 0 to 3
STEAM	Stimulated Echo Acquisition Mode
STROBE	Strengthening the Reporting of Observational Studies in Epidemiology
TE	Echo Time
TR	Repetition Time
